# Evaluation of Gamma Aminobutyric Acid and Sodium Butyrate in Juvenile Red Seabream (*Pagrus major*) Diets Containing Graded Levels of Fish Meal and Soy Protein Concentrate

**DOI:** 10.3390/ani14131973

**Published:** 2024-07-03

**Authors:** Buddhi E. Gunathilaka, Seong-Mok Jeong, Kang-Woong Kim, Seunghan Lee, Sang-Woo Hur, Sang-Guan You, Sang-Min Lee

**Affiliations:** 1 Department of Aquatic Life Medicine, Gangneung-Wonju National University, Gangneung 25457, Republic of Korea; glbeg44@gwnu.ac.kr; 2 Aquafeed Research Center, National Institute of Fisheries Science, Pohang 37517, Republic of Korea; smjeong1@korea.kr (S.-M.J.);; 3 Department of Marine Food Science and Technology, Gangneung-Wonju National University, Gangneung 25457, Republic of Korea; umyousg@gwnu.ac.kr

**Keywords:** gamma aminobutyric acid, sodium butyrate, red seabream, functional ingredient, fish meal replacement, soy protein concentrate

## Abstract

**Simple Summary:**

Fish meal replacement in aquafeed is a critical management practice in sustainable aquaculture to minimize the use of marine-origin resources while improving production. Gamma aminobutyric acid and sodium butyrate are functional ingredients used in aquafeed. Both ingredients were reported to improve the growth performance, feed efficiency and health status of fish species, although they have not been studied in red seabream (*Pagrus major*) diets. In this study, we investigated the supplementary effects of gamma aminobutyric acid and sodium butyrate when a graded level of fish meal was replaced with soy protein concentrate in diets focusing on growth performance, feed utilization, serum lysozyme activity, plasma biochemical parameters and muscle proximate composition. The results showed that the growth performance of red seabream was improved after dietary supplementation with both additives. Feed utilization was improved in fish fed on diets containing low fish meal levels with sodium butyrate. Both additives improved the lysozyme activity of fish at each fish meal replacement level. Plasma total cholesterol and total protein levels were significantly reduced due to fish meal replacement. Therefore, these findings suggest that gamma aminobutyric acid and sodium butyrate are potentially functional ingredients to use in red seabream diets even after substantially replacing fish meal with soy protein concentrate.

**Abstract:**

The experiment was conducted to evaluate the supplementary effects of gamma aminobutyric acid (GABA) and sodium butyrate (SB) when a graded level of fish meal (FM) was replaced with soy protein concentrate (SPC) in diets for juvenile red seabream (*Pagrus major*). A control diet was designed to contain 60% FM (F60). Two other diets were formulated by reducing FM levels to 40% and 20% with SPC (F40 and F20). Six more diets were formulated by adding 0.02% GABA or 0.2% SB to each F60, F40 and F20 diets (F60G, F60S, F40G, F40S, F20G and F20S). Each diet was randomly assigned to a triplicate group of fish (5.52 g/fish) and provided for eight weeks. Final body weight, weight gain and specific growth rate of fish fed F60G, F60S, F40G and F40S diets were comparable and significantly higher (*p* < 0.05) than other groups. The growth of fish fed SB-containing diets was significantly increased (*p* < 0.05) compared to fish fed the respective control diets. The feed efficiency and protein efficiency ratios were significantly higher (*p* < 0.05) in the fish fed all diets containing 60% and 40% FM compared to F20 and F20G groups. The F40S diet resulted in the highest feed utilization values. The F20S group exhibited significantly higher (*p* < 0.05) feed utilization than the F20 and F20G groups. Serum lysozyme activity was significantly higher (*p* < 0.05) in fish fed the GABA- and SB-containing diets compared to the F20 group. The F60S group exhibited the highest lysozyme activity which was significantly higher (*p* < 0.05) than in the F20 and F40 groups. Therefore, the growth performance, feed utilization and innate immunity of red seabream can be enhanced by dietary supplementation with GABA or SB in low-FM diets containing SPC. The FM level in the juvenile red seabream diet can be reduced to 40% with SPC and GABA or SB while maintaining performance better than a diet containing 60% FM.

## 1. Introduction

Gamma aminobutyric acid (GABA) is a non-protein amino acid necessary for inhibitory neurotransmission, one of the major physiological functions in animals. GABA has been evaluated as a dietary supplement for different fish species including fresh water, brackish water and marine water species representing herbivorous, omnivorous and carnivorous feeding habits. GABA was reported to improve growth performance, innate immunity, gut function, digestive enzyme activities, feed utilization, hypoxia tolerance and disease resistance in fish species as a feed additive [1,2,3,4]. The supplement level of GABA was also reported to range between 0.01% and 1% in diets for different fish species including 0.5–1.0% for Indian major carp, mrigal (*Cirrhinus mrigala*) [1], 0.02% (237 mg/kg) for olive flounder (*Paralichthys olivaceus*) [2,4] and 0.2–0.5% for Nile tilapia (*Oreochromis niloticus*) [3].

Sodium butyrate (SB), the sodium salt of butyric acid, is also a functional ingredient used in aquafeed. It is used as a single feed additive or as a compound in mixtures of other short- or medium-chain carboxylic acid salts in aquafeeds [5,6]. Butyrate is also supplied as a form of butyric acid glycerides (butyrin) in aquafeeds [7,8]. SB is well documented to improve the growth performance and feed utilization efficiency of many aquaculture species. Interestingly, it improves the gut health and digestive enzyme activities in fish [9,10,11,12] and alleviates the negative impacts of high-fat diets on liver and gut microbiota [13,14] while improving the resistance of fish to heat stress [15]. 

Fish meal (FM) replacement in aquafeed is a critical management practice for sustainable aquaculture which minimizes the use of marine-origin resources while improving production [16]. In this regard, GABA and SB or other butyrate forms have been evaluated in low-FM diets for several fish species and both additives were reported to enhance the efficiency of low-FM diets. GABA prevented growth impairment and intestinal malfunctions in turbot (*Scophthalmus maximus*) caused by high soybean meal diets [17]. SB is especially reported to protect the fish gut from impairments caused by high levels of soybean meal in diets. For instance, intestinal enteropathy induced by soybean meal was alleviated in turbot by dietary SB by improving the intestinal tight junction, suppressing inflammation and altering the microbial community [18]. Gut health was improved through enhanced anti-inflammatory activities and gut microbiota in largemouth bass (*Micropterus salmoides*) fed a diet containing a high level of soybean meal [19]. The feed conversion and distal intestine morphology of the yellow drum (*Nibea albiflora*) was also enhanced by SB after feeding a low-FM diet containing soybean meal [20].

Red seabream is an important fish species cultured in South Korea. The effects of GABA, SB or other butyrate forms have not been evaluated in red seabream diets to the best of our knowledge. However, SB or other functional ingredients containing butyrate were evaluated in fish species belonging to the seabream family (Sparidae). Growth performance and nutrient digestion were increased in gilthead seabream (*Sparus aurata*) fed butyrate [21]. Two other studies revealed that SB improves the metabolic steady status of gilthead seabream fed diets containing low levels of FM and fish oil [22,23]. Microencapsulated SB resulted in enhanced body growth, intestinal development and antioxidative activity of juvenile black seabream (*Acanthopagrus schlegelii*) when incorporated at 0.2% in diets [24]. A mixture of dietary mono-, di- and tributyrin also improved the digestive enzyme activities of black seabream fed diets containing high levels of soybean meal [7]. Zarei et al. [25] evaluated increasing the levels of dietary butyric acid glycerides in yellowfin seabream diets (*Acanthopagrus latus*) and reported positive influences on immunity, growth and liver antioxidant activities. The replacement of FM in the red seabream diet has been a well-studied research area over the last few decades. Different types of alternative protein sources were evaluated as FM replacers and most of these studies revealed potential ingredients to replace FM in the red seabream diet. Soy protein concentrate (SPC) is one of the successful protein ingredients used in red seabream diets [26], although high SPC levels in diets resulted in growth impairments. Protein sources that were developed, i.e., protein hydrolysates and functional ingredients, were successfully applied to alleviate the impairments caused by high SPC levels in diets [27,28,29]. Therefore, considering FM replacement and the aforementioned positive influences of both GABA and SB, we designed the present study to evaluate the effects of GABA and SB on growth performance, feed utilization, serum lysozyme activity, plasma biochemical parameters and muscle proximate composition when graded levels of FM were replaced with SPC in diets for juvenile red seabream.

## 2. Materials and Methods

### 2.1. Experimental Diets

**Figure 1:**
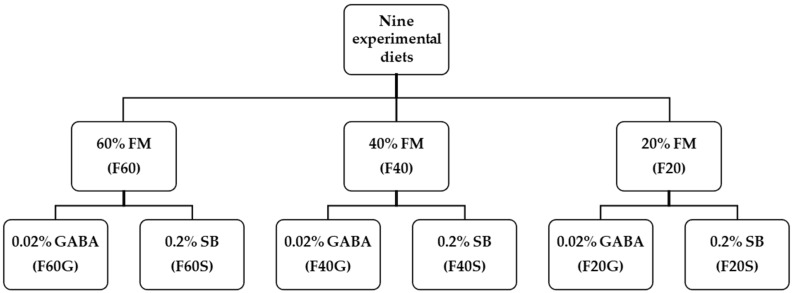
A self-explanatory figure of experimental diets. A total of nine diets were formulated having three different fish meal (FM) levels. Three diets were designed to contain FM at 60% (FM60), 40% (FM40) or 20% (FM20), respectively. Each diet containing three FM levels received added gamma aminobutyric acid (GABA) at 0.02% or sodium butyrate (SB) at 0.2% to prepare six different experimental diets (designated as F60G, F60S, F40G, F40S, F20G and F20S, respectively).

**Figure 2:**
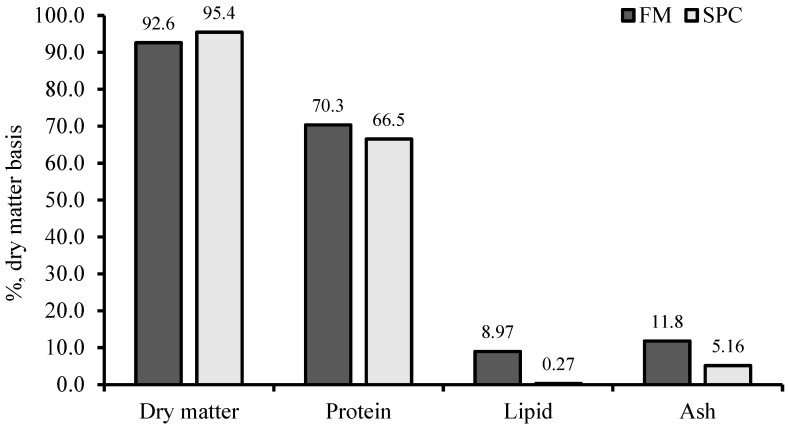
Proximate composition (%, dry matter basis) of fish meal (FM) and soy protein concentrate (SPC).

**Figure 3:**
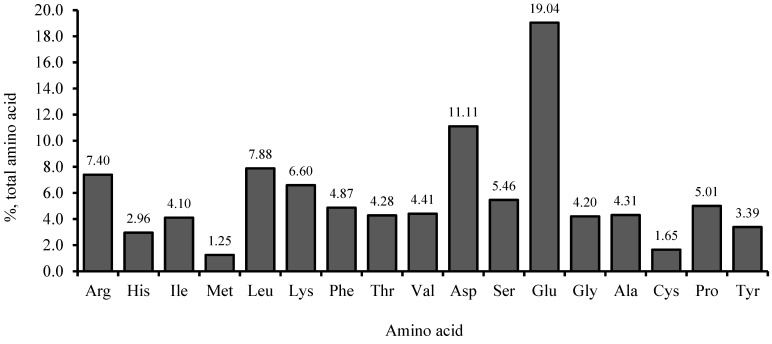
Amino acid composition of soy protein concentrate (%, total amino acids).

Nine diets were formulated for the experiment to contain 45% crude protein and 14% crude lipid, as shown in Figure 1 and Table 1. A diet formulated to contain 60% FM was considered the control (F60). Two other diets were formulated by reducing FM levels to 40% and 20% with SPC (FM40 and FM20). Then, six more diets were formulated by adding 0.02% GABA or 0.2% SB to each F60, FM40 and FM20 diets (designated as F60G, F60S, F40G, F40S, F20G and F20S, respectively). Inclusion level of GABA was decided according to previously recorded optimum levels for carnivorous fish species [2] and SB level was decided according to published data for fish species in the same Sparidae family [24]. Dry ingredients of each diet were then mixed by adding the required amount of fish oil, according to feed formulation, and distilled water in order to making doughs. Each dough was extruded using a noodle machine, crushed to a size of 3–5 mm and dried at 35 °C for 12 h creating sinking dry pellets. Dry diets were kept at −20 °C till the feeding trial began. Proximate composition of FM and SPC are presented in Figure 2. Amino acid levels of SPC are provided in Figure 3.

### 2.2. Experimental Fish Feeding Trial and Conditions

Juvenile red seabream were provided by a private hatchery (Uljin county, Korea). They were then stocked at the Research Facilities of Gangneung-Wonju National University, Marine Biology Center. Following an acclimation period for two weeks, juveniles, averaging 5.52 ± 0.02 g, were stocked at a density of 25 fish per tank in 27 tanks (100 L). Nine diets were divided into three replicates, and each tank was randomly allocated to one of them. Fish were fed on experimental diets until satiation (twice a day, 09:00 and 17:00 h) for eight weeks. The photoperiod was adjusted to match the season’s actual day duration as precisely as possible. Water temperature (17.7–21.3 °C), pH (7.47–7.87), and dissolved oxygen (6.57–7.29 mg/L) were recorded daily.

### 2.3. Sample Collection and Analyses

At the end of the feeding trial, fish were fasted for 24 h, counted, and weighed for the calculation of survival, growth, and feed utilization efficiency parameters including weight gain (WG), specific growth rate (SGR) feed intake (FI), feed efficiency (FE) and protein efficiency ratio (PER). Three fish were randomly sampled from each tank and stored frozen at −20 °C for analysis of muscle proximate composition. Blood samples were collected from six individual fish from each experimental tank using heparinized syringes to separate the plasma for subsequent hematological analyses. Another six fish were captured and blood samples collected with non-heparinized syringes to separate serum samples for analysis of non-specific immune parameters. Both plasma and serum were separated by centrifugation at 5000× *g* for 10 min and stored at −70 °C. Blood samples were allowed to clot at room temperature for 30 min prior to separating serum samples. Remaining fish in all tanks were sedated separately, total lengths were measured to closest 0.1 mm and they were dissected to weigh viscera and livers to calculate condition factor (CF) and organosomatic indices. All the fish were sedated with 200 ppm 2-phenoxyethanol before sampling according to ethical guidelines (GWNU-2022-14).

Standard procedures were followed to assess the moisture and ash content of fish muscle samples and experimental diets [30]. A Kjeltec Analyzer (Buchi, Flawil, Switzerland) was used to test crude protein, and a Soxhlet extractor (VELP Scientifica, Milano, Italy) was used to analyze crude lipid. Amino acid compositions were analyzed using automatic amino acid analyzer (Hitachi, Tokyo, Japan). Lysozyme activity of serum samples was measured on the basis of turbidimetric technique with *Micrococcus lysodeikticus* (Sigma, St. Louis, MO, USA) as a substrate [31]. Superoxide dismutase activity (SOD) of serum samples was analyzed with an assay kit (Sigma 19,160). An automated blood analyzer (FUJI DRI-CHEM NX500i, FUJiFILM Corporation, Tokyo, Japan) was used to measure plasma glutamic-oxaloacetic transaminase, alkaline phosphatase, total cholesterol, triglycerides, total protein and glucose levels using assay kits (reference code: 3150, 3550, 1450, 1650, 1850 and 2050, respectively) purchased from FUJiFILM Co., Tokyo, Japan.

### 2.4. Statistical Analysis

Data were analyzed using one-way analysis of variance. Duncan’s multiple range test [32] was used to determine the significant differences in the mean values of analyzed parameters, using SPSS version 20.0 (SPSS Inc., Chicago, IL, USA). Then, significance due to levels of FM, GABA, SB or their interaction was analyzed using two-way analysis of variance. Statistical significance was determined at *p* < 0.05. Data were presented as mean ± standard deviation. Arcsine transformation was performed on percentage data prior to statistical analysis.

## 3. Results

### 3.1. Growth Performance

The growth performance, feed utilization and survival of the red seabream fed experimental diets are presented in Table 2. At each FM-replacement level, those fish fed diets containing GABA and SB exhibited high growth performance compared to the respective control groups. The FBW of fish fed F60G, F60S and F40S diets was significantly increased compared to that of fish fed other experimental diets except F40G group (*p* < 0.05). The fish fed F60 and F40 diets exhibited comparable results which were significantly higher than diets containing 20% FM and significantly lower than F60G, F60S and F40S diets (*p* < 0.05). The F20 group exhibited the lowest FBW which was significantly lower than all other groups (*p* < 0.05). WG was significantly higher in F60S and F40S groups compared to that of fish fed F60, F40 and F20 diets including F20G and F20S (*p* < 0.05). The WG observed in the F40 group was significantly lower than that of the F60G and F60S groups (*p* < 0.05). The WGs of fish fed F60, F40 and F40G diets were comparable. Fish fed F20 and F20G diets exhibited significantly lower WGs compared to the F20S group (*p* < 0.05). However, all diets containing 20% FM resulted in significantly lower WG compared to other diets containing 60% or 40% FM levels (*p* < 0.05). SGR was significantly higher in the F60S group compared to that of the F60, F40, F20, F20G and F20S groups (*p* < 0.05). The SGR of fish fed the F40S diet exhibited significantly higher results compared to that of F40 F20, F20G and F20S groups (*p* < 0.05). The SGRs of F60, F60G, F40G and F40S were comparable. The SGRs observed in F60, F40 and F40G were also comparable. Fish fed F20 and F20G diets exhibited significantly lower SGRs compared to the F20S group and fish fed all diets containing 20% FM resulted in significantly lower SGRs compared to other groups (*p* < 0.05). The FM replacement level, GABA and SB supplementation significantly affected the FBW, WG and SGR. However, their interactions did not significantly affect fish growth performance.

### 3.2. Feed Utilization and Survival

The FI of fish was significantly affected (*p* < 0.05) by dietary FM level, GABA and SB although their interaction was not significantly different. The highest FE and PER were observed in fish fed the F40S diet. The FE and PER of fish fed all other diets containing 60% and 40% FM exhibited comparable results to those of F40S and F20S groups. The FE and PER of fish fed F20 and F20G diets were significantly lower compared to the F20S group (*p* < 0.05). Both FE and PER were significantly affected by FM replacement, SB supplementation and their interaction (*p* < 0.05). However, dietary GABA or its interaction with FM level did not significantly affect the FE and PER. The survival of fish was also not significantly affected by dietary treatments.

### 3.3. Non-Specific Immune Responses

The non-specific immune responses of red seabream fed the experimental diets are presented in Table 3. The lysozyme activity of the fish fed F60S diets exhibited significantly higher values compared to that of F40 and F20 groups. The lysozyme activity in the F20 group was comparable with that of the F40 group and significantly lower than all other groups. Lysozyme activity was also significantly affected by FM replacement and dietary SB supplementation. GABA and the interactions of both GABA and SB with FM level were not significantly different. SOD activity was not significantly affected by dietary treatments.

### 3.4. Plasma Biochemical Parameters

Plasma biochemical parameters of juvenile red seabream fed the nine experimental diets are shown in Table 4. Plasma total cholesterol level was significantly higher in the F60S group compared to fish fed diets containing 40% and 20% FM (*p* < 0.05). The total cholesterol level was significantly affected by the FM level in the diets by decreasing the concentration when the FM level was reduced in the diet, although neither GABA nor SB affected it significantly (*p* < 0.05). The total protein level was also significantly affected by the FM levels in diets (*p* < 0.05). The total protein level was significantly higher in F60G and F60S groups compared to that of the fish fed on diets containing 20% FM (*p* < 0.05). Plasma glutamic-oxaloacetic transaminase, alkaline phosphatase, triglyceride and glucose levels were not significantly affected by dietary treatments.

### 3.5. Biometric Parameters

The biometric parameters of red seabream fed on the experimental diets are presented in Table 5. CF, HSI and VSI were not significantly different among the fish fed on experimental diets. However, HSI was significantly affected by the interaction of FM level and SB (*p* < 0.05). VSI was significantly affected by SB (*p* < 0.05).

### 3.6. Muscle Proximate Composition

The muscle proximate composition of red seabream fed the nine experimental diets is presented in Table 6. The muscle proximate composition was not significantly altered by the experimental diets. Neither did FM replacement, GABA and SB supplementation or their interaction with FM level have a significant influence on muscle proximate composition.

## 4. Discussion

The efficiency of aquafeed is mainly represented by the growth performance and feed utilization of fish. The growth performance of red seabream was significantly reduced due to dietary FM replacement in the present study. GABA and SB improved fish growth performance in each FM replacement level compared to the respective control groups. Both GABA and SB are well documented as growth promoters of fish species. GABA enhanced the intestinal morphology and the expression of growth-related genes, GH, GHR2, IGF-I and IGF-II, in Nile tilapia [3]. It upregulated the expression of ghrelin and IGF-I genes in rohu (*Labeo rohita*) [33]. Wu et al. [34] revealed that GABA improves the expression of feeding-related genes in grass carp (*Ctenopharyngodon idellus*). Dietary SB was also reported to enhance the expression of ghrelin, GH and IGF-1 in Nile tilapia [11]. It improves the intestine absorption capacity of grass carp by accelerating the expression of peptide transporter 1 gene [12]. SB accelerated the activities of digestive enzymes in several fish species fed diets containing a similar FM level [35,36]. Therefore, these positive influences of GABA and SB as fish growth promoters could be responsible for improved growth performance at each FM replacement level.

The WG and SGR of fish fed F40G and F20G diets were statistically comparable with fish fed the respective control diets (F40 and F20). The fish fed the F40G diet fully restored the performances of the diets containing 60% FM. Li et al. [17] observed significantly higher growth performance in turbot fed a high soybean meal with GABA. They also observed that GABA ameliorates the adverse effects of a high soybean meal diet on the turbot intestine as the main reason for restored growth performance, in addition to increased FI. However, the effects of GABA were not well studied in carnivorous fish fed low-FM diets. We used SPC as the FM replacement in the red seabream diet in the present study. It is well documented that high levels of dietary SPC retarded the growth performance of red seabream [37,38]. FI was not significantly increased in fish groups fed diets containing GABA compared to the respective control groups, indicating that growth was not improved due to FI in the present study. SPC contains a lesser amount of antinutritional factors due to its processing technology compared to soybean meal. Therefore, the growth inhibitory effect of SPC might be lower, resulting in sufficient growth in fish fed on diets containing 40% and 20% FM without GABA or SB. On the other hand, several studies reported an optimal level of GABA in diets for better performance in fish [33,39]. In the current study, the inclusion level of GABA was decided according to previously reported optimum levels for other carnivorous fish species [2]. The optimum level can be different from one fish species to another even in fish species classified in the same family. Therefore, we suggest that an optimum level of GABA should be found for red seabream diets containing low-FM levels. Further studies should also be conducted to reveal the effects of GABA on the growth performance of fish fed low-FM diets.

The growth performance of fish fed low-FM diets containing SB exhibited significantly higher results than the respective control groups. The F40S diet fully replicated the performance of the fish fed diets containing 60% FM. Several studies reported that SB improves the growth performance and feed utilization of fish fed low-FM diets. Liu et al. [40] reported that the digestive enzyme activities, intestinal morphology, disease resistance and gut microbiota of rainbow trout was improved after feeding a low-FM diet containing SB. Liu et al. [18] observed that SB improves the intestinal tight junction, microbial composition and anti-inflammatory activity while alleviating soybean-induced enteropathy in turbot fed high-soybean meal diets. In largemouth bass, dietary SB improved the gut health through reduced inflammation and improved gut morphology when supplemented in a high soybean meal diet [41]. SB also improved the expression of tight junction protein and the intestinal structure of yellow drum (*Nibea albiflora*) fed high soybean meal diets [20]. Therefore, these types of positive influences might be considered as reasons for the significantly improved growth performance and feed utilization in fish fed diets containing SB compared to the respective control diets. In particular, restored feed utilization might be attributed to its effects on gut morphology, digestive enzyme activities and intestine microbiota. Future studies should be conducted to evaluate the effects of SB on the gut functions of red seabream.

Serum lysozyme activity was evaluated in all treatment groups as an indicator of innate immune functions. Serum lysozyme activity was significantly higher in the F60S group compared to the F40 and F20 groups. All the groups exhibited significantly higher results than the F20 group except for F40. Fish fed GABA- and SB-containing diets resulted in higher activity compared to the respective control diets containing similar FM levels, indicating the positive influence of both additives on innate immunity. The F20G and F20S groups exhibited significantly higher results compared to the F20 group, highlighting the restorative role of GABA and SB on retarded immunity due to FM replacement. SOD activity was slightly improved by dietary GABA at 40% and 20% FM-replacement levels although the results were not significantly different. Temu et al. [39] observed significant improvement in SOD level in Nile tilapia when they were fed diets containing GABA, although the SOD activity was not significantly enhanced due to GABA in the present study. Li et al. [17] observed improved antioxidant capacity in turbot fed a low-FM diet containing GABA. They suggested that GABA alleviated oxidative stress caused by high soy protein through direct involvement during lipid peroxidation, as observed by Deng et al. [42]. SOD activity in serum and lysozyme activity in head kidney and spleen of Jian carp (*Cyprinus carpio* var. Jian) were significantly improved by dietary GABA [19]. They assumed that GABA stimulates the immune system of fish as a reason for the results. In line with present results, Farris et al. [2] reported that dietary GABA significantly improved serum lysozyme activity of olive flounder while SOD activity was not significantly affected. They assumed the GABA-associated macrophage activation in the immune system, according to Kim et al. [43], as the reason for improved lysozyme activity. In phagocytes, GABA participates in the GABAergic signaling process that leads to accelerated phagocytic activity [44]. Lysozyme is one of the main enzymes utilized in phagocytic activity. Therefore, in our study, lysozyme activity in red seabream might be improved by dietary GABA, since these mechanisms have been observed to enhance activities compared to control groups. In the case of SB, the lysozyme activity of grey mullet (*Liza ramada*) was significantly improved by dietary SB supplementation [36]. They suggested that role of SB in immune stimulation and protection against lipid peroxidation to be the reason. Dawood et al. [15] observed that the lysozyme activity of Nile tilapia was improved by dietary SB even after being subjected to heat stress. They assumed the improved activity of the intestinal microbiota was the reason for this observation. SB also exhibits the ability to reduce inflammatory responses in several fish species [10]. Consequently, the immune responses of red seabream might be improved by SB in the present study.

Plasma biochemical parameters are important for understanding the health status and physiological condition of fish. Cholesterol and TP concentrates of red seabream plasma were significantly influenced by dietary treatments. Both parameters exhibited significant interaction with FM level or SPC level in diets regardless of the GABA or SB. In a previous study, we observed that different protein sources including SPC, alone or mixed with other protein sources, had no significant influence on plasma cholesterol or TP levels when each diet contained similar FM levels [45]. A similar trend was observed by Biswas et al. [46]. In contrast, neither parameter was significantly affected when a graded level of FM was replaced with fermented rapeseed meal in the juvenile red seabream diet [47]. Kader et al. [48] also observed no significant influence on the blood cholesterol level of red seabream fed diets with different levels of FM replaced with dehulled soybean meal. However, the TP level was significantly increased with an increase in dehulled soybean meal in diets. Biswas et al. [49] reported no significant changes in plasma cholesterol level of red seabream fed decreasing levels of SPC. The biochemical parameters of red seabream are dependent on different factors including environmental conditions and immune status, in addition to feed [50,51]. Therefore, these discrepancies with the results of the previous studies might be attributed to such factors. The nutrient content of diets, mainly the amino acid and fatty acid levels, was changed with the level of feed ingredients [45]. High dietary SPC levels affected the somatic indexes, gut enzyme activities and lipid accumulation in red seabream [37]. These changes can influence the nutrient absorption and metabolism of fish [52]. Accordingly, different combinations of FM and SPC in diets might have significant effects on the plasma cholesterol and TP levels in the red seabream used in the present study.

The CF, HSI and VSI were not significantly different among the dietary groups. However, the interaction of FM and SPC levels with SB exhibited a significant impact on HSI. SB showed a significant influence on VSI according to two-way ANOVA. HSI and VSI indicate the weight of liver and viscera compared to body weight. In accordance with aforementioned evidence from previous studies, SB improves intestinal structure, nutrient absorption, microbial community and health status while suppressing intestinal inflammation [18,41]. Accordingly, we assumed that HSI and VSI were improved due to those influences which might enhance the growth of fish fed SB-containing diets compared to each control group with different FM replacement levels. Muscle proximate compositions were also not significantly changed by the different FM or SPC levels, GABA and SB supplementation in diets. A number of previous studies have observed similar results when fish were fed diets containing similar protein and lipid levels [6,22]. The dietary composition of fatty acid, amino acid and other compounds, i.e., vitamins, was documented to reflect in the muscle, whole-body or liver samples [45,53]. Therefore, further studies should be conducted to evaluate the effects of nutrient levels in diets containing SB or GABA on the muscle, liver and whole-body composition of red seabream.

A high level of FM replacement resulted in lower performance according to most analyzed or calculated parameters in the present study. We evaluated F20 diets containing 20% FM and approximately 40% SPC as the highest FM replacement level. However, the growth performance of the control diet was not replicated even after incorporating GABA or SB. Several studies reported that some measure of FM could be replaced in red seabream diets with SPC. Hossain et al. [28] reduced the FM level in same-sized red seabream to 15% with approximately 40% SPC, lysine, methionine and inosine monophosphate while restoring growth performance, immune responses and nutrient digestibility of diet. Biswas et al. [46] (2017) observed restored growth of juvenile red seabream (3.9 g) when FM level was reduced to 30% with 30% SPC, 10% corn gluten and taurine compared to a control diet containing 70% FM. Biswas et al. [26] reduced the FM level to 20% with 40% SPC, 10% corn gluten and taurine replicating the performance of red seabream (23 g) fed a control diet containing 67% FM. These previous findings are slightly inconsistent with the results of the present study indicating influences from other factors by which the growth performance of red seabream was either accelerated or decreased. Experimental feed formulated by Hossain et al. [28] contained lysine and methionine in addition to inosine monophosphate. The studies conducted by Biswas et al. [26,46] formulated diets to contain approximately 13% crude ash, lecithin and 1% taurine without lysine and methionine. Water temperatures were also different among these studies. Therefore, the discrepancy can be attributed to these differences, including feed formulations, environmental conditions and fish growth stages. We suggest that the level of functional feed additives such as lysine, methionine, taurine, mineral and vitamin levels should be increased in low-FM diets in our formulation to enhance the effectiveness of F20 diets. Moreover, the trial period might also be one of the main reasons for the discrepancies. For instance, the growth performance of red seabream was not restored after a 15-week feeding trial when FM level was reduced from 40% to 25% with SPC [6]. Future studies should be conducted to elucidate these matters and an appropriate FM level in between 40% and 20% should be found in diets containing SPC with GABA or SB.

## 5. Conclusions

In summary, GABA seems to improve the performance of red seabream fed diets containing high SPC levels. SB improved the growth performance and feed utilization of fish fed high-SPC diets. Therefore, the precise supplement level of GABA and SB should be evaluated in future studies. Both GABA and SB should be investigated for possible synergistic effects in low-FM diets. The present results also suggested that FM replacement level should be adjusted between 40% and 20% in future studies. In general, the FM level in the juvenile red seabream’s diet can be reduced to 40% with SPC and GABA or SB while maintaining performance better than a diet containing 60% FM.

## Figures and Tables

**Table 1:** Formulation and proximate composition of the experimental diets for red seabream (*Pagrus major*) (%, dry matter basis).

Ingredients	F60	F60G	F60S	F40	F40G	F40S	F20	F20G	F20S
Fish meal (FAQ 65) ^1^	60.0	60.0	60.0	40.0	40.0	40.0	20.0	20.0	20.0
GABA ^2^		0.02			0.02			0.02	
Sodium butyrate ^3^			0.20			0.20			0.20
Soy protein concentrate	3.75	3.75	3.75	20.10	20.10	20.10	38.45	38.45	38.45
Corn gluten meal				2.00	2.00	2.00	2.00	2.00	2.00
Wheat flour	20.0	20.0	20.0	20.0	20.0	20.0	20.0	20.0	20.0
Fish oil	1.00	1.00	1.00	3.00	3.00	3.00	4.95	4.95	4.95
Soybean oil	5.78	5.78	5.78	5.49	5.49	5.49	5.24	5.24	5.24
Mineral mix ^4^	1.00	1.00	1.00	1.00	1.00	1.00	1.00	1.00	1.00
Vitamin mix ^5^	1.00	1.00	1.00	1.00	1.00	1.00	1.00	1.00	1.00
Starch	6.97	6.95	6.77	4.51	4.49	4.31	2.60	2.58	2.40
Choline chloride	0.50	0.50	0.50	0.50	0.50	0.50	0.50	0.50	0.50
Lysine				0.34	0.34	0.34	0.63	0.63	0.63
Methionine				0.49	0.49	0.49	1.00	1.00	1.00
Taurine				0.07	0.07	0.07	0.13	0.13	0.13
Monocalcium phosphate				1.50	1.50	1.50	2.50	2.50	2.50
Proximate composition									
Dry matter	93.6	93.3	94.2	93.3	93.6	94.3	93.8	93.7	95.1
Protein	46.4	47.1	47.9	46.1	46.3	46.0	47.6	47.2	46.5
Lipid	13.3	14.3	14.0	13.8	13.7	14.3	14.6	14.7	14.2
Ash	11.1	11.2	11.3	10.0	9.83	10.0	8.73	8.34	8.53

^1^ Danish fish meal. ^2^ Gamma aminobutyric acid (99%), Sigma-Aldrich, St. Louis, MO, USA. ^3^ 98%, Sigma-Aldrich, Steinheim, Germany. ^4^ Vitamin mixture composition (unit/kg mix): ascorbic acid, 6400 mg; tocopherol acetate, 37,500 mg; thiamin nitrate, 5000 mg; riboflavin, 10,000 mg; pyridoxine hydrochloride, 5000 mg; nicotinic acid, 37,500 mg; Ca-D-pantothenate, 17,500 mg; inositol, 75,000 mg; biotin, 50 mg; folic acid, 2500 mg; menadione sodium bisulfite, 2500 mg; retinol acetate, 5,000,000 IU; cholecalciferol, 1,000,000 IU; cyanocobalamin, 25 mg; riboflavin, 10,000 mg. ^5^ Mineral mixture composition (g/kg mix); ferrous fumarate, 12.5; manganese sulfate, 11.3, ferrous sulfate, 20; cupric sulfate, 1.25; cobaltous sulfate, 0.75; zinc sulfate, 13.75; calcium iodate, 0.75; magnesium sulfate, 80.2; aluminum hydroxide, 0.75.

**Table 2:** Growth performance and feed utilization and survival of red seabream (*Pagrus major*) fed the nine experimental diets for 8 weeks.

	FBW (g) ^1^	WG (%) ^2^	SGR (%) ^3^	FI (g/fish) ^4^	FE (%) ^5^	PER ^6^	SUR (%) ^7^
F60	19.5 ± 1.20 ^b^	255 ± 19.9 ^bc^	2.26 ± 0.10 ^bc^	22.3 ± 1.52	59.4 ± 0.46 ^ab^	1.28 ± 0.01 ^ab^	88.0 ± 4.00
F60G	21.0 ± 0.51 ^a^	281 ± 18.3 ^ab^	2.39 ± 0.09 ^ab^	23.5 ± 1.81	61.9 ± 8.42 ^ab^	1.31 ± 0.18 ^ab^	84.0 ± 4.00
F60S	21.4 ± 0.29 ^a^	287 ± 9.56 ^a^	2.42 ± 0.04 ^a^	24.1 ± 0.43	61.4 ± 2.50 ^ab^	1.28 ± 0.05 ^ab^	84.0 ± 4.00
F40	19.1 ± 0.23 ^b^	247 ± 0.67 ^c^	2.22 ± 0.00 ^c^	20.8 ± 0.50	59.2 ± 2.75 ^ab^	1.28 ± 0.06 ^ab^	81.3 ± 2.31
F40G	20.1 ± 0.53 ^ab^	265 ± 6.49 ^abc^	2.31 ± 0.03 ^abc^	23.1 ± 1.82	59.0 ± 5.26 ^ab^	1.27 ± 0.11 ^ab^	84.0 ± 4.00
F40S	21.1 ± 0.62 ^a^	285 ± 21.2 ^a^	2.40 ± 0.10 ^ab^	22.5 ± 1.39	66.0 ± 5.26 ^a^	1.44 ± 0.11 ^a^	88.0 ± 10.6
F20	14.0 ± 0.47 ^e^	154 ± 15.8 ^e^	1.66 ± 0.11 ^e^	19.0 ± 1.43	38.6 ± 2.02 ^c^	0.84 ± 0.04 ^c^	82.7 ± 4.62
F20G	15.3 ± 0.34 ^d^	176 ± 7.28 ^e^	1.81 ± 0.05 ^e^	21.4 ± 1.19	39.7 ± 4.08 ^c^	0.86 ± 0.09 ^c^	81.3 ± 2.31
F20S	17.4 ± 1.30 ^c^	216 ± 21.7 ^d^	2.05 ± 0.12 ^d^	19.9 ± 1.48	56.4 ± 3.93 ^b^	1.23 ± 0.09 ^b^	89.3 ± 10.1
Two-way ANOVA (*p* value)							
FM level	0.000	0.000	0.000	0.022	0.000	0.000	0.169
GABA	0.001	0.004	0.004	0.013	0.616	0.746	0.615
SB	0.000	0.000	0.000	0.025	0.000	0.000	0.346
FM × GABA	0.778	0.852	0.787	0.748	0.877	0.926	0.317
FM × SB	0.235	0.312	0.092	0.769	0.003	0.002	0.320

Values are the mean of triplicate groups and are presented as mean ± SD. Values with different superscripts in the same column are significantly different (*p* < 0.05). The lack of superscript letters indicates no significant differences among treatments. ^1^ Final mean body weight. ^2^ Weight gain = 100 × (final mean body weight − initial mean body weight)/initial mean body weight. ^3^ Specific growth rate = [(ln final body weight − In initial body weight)/days] × 100. ^4^ Feed intake = dry feed consumed (g)/number of fish. ^5^ Feed efficiency = (fish wet weight gain/feed intake) × 100. ^6^ Protein efficiency ratio = wet weight gain/total protein given. ^7^ Survival rate = (final fish number/initial fish number) × 100.

**Table 3:** Non-specific immune response of red seabream (*Pagrus major*) fed the nine experimental diets for 8 weeks.

	Lysozyme ^1^	SOD ^2^
F60	29.6 ± 0.81 ^ab^	76.7 ± 5.38
F60G	30.0 ± 0.85 ^ab^	75.2 ± 1.62
F60S	30.7 ± 0.49 ^a^	79.2 ± 4.64
F40	27.8 ± 0.96 ^bc^	72.6 ± 2.87
F40G	29.1 ± 1.03 ^ab^	75.3 ± 1.50
F40S	29.3 ± 0.49 ^ab^	76.3 ± 1.11
F20	26.3 ± 0.37 ^c^	72.0 ± 1.42
F20G	28.5 ± 0.67 ^ab^	75.3 ± 0.56
F20S	29.1 ± 0.37 ^ab^	76.6 ± 0.75
Two-way ANOVA (*p* value)		
FM level	0.036	0.668
GABA	0.055	0.519
SB	0.004	0.203
FM × GABA	0.538	0.630
FM × SB	0.402	0.945

Values are means of triplicate groups and presented as mean ± SD. Values with different superscripts in the same row are significantly different (*p* < 0.05). The lack of superscript letters indicates no significant differences among treatments. ^1^ Lysozyme activity (µg mL^−1^). ^2^ Superoxide dismutase (% inhibition).

**Table 4:** Plasma biochemical parameters of juvenile red seabream (*Pagrus major*) fed the nine experimental diets for 8 weeks.

	GOT (U/L) ^1^	ALP (U/L) ^2^	T-CHO (mg/dL) ^3^	TG (ng/dL) ^4^	TP (g/dL) ^5^	GLU (mg/dL) ^6^
F60	33.0 ± 0.82	141 ± 73.5	241 ± 6.53 ^ab^	344 ± 128	3.15 ± 0.29 ^ab^	110 ± 25.0
F60G	39.0 ± 1.63	231 ± 55.2	263 ± 6.74 ^ab^	165 ± 12.7	3.47 ± 0.09 ^a^	134 ± 28.8
F60S	30.0 ± 0.82	222 ± 108	296 ± 15.9 ^a^	273 ± 52.3	3.37 ± 0.07 ^a^	113 ± 8.37
F40	42.0 ± 3.61	186 ± 24.5	216 ± 15.6 ^bc^	326 ± 90.7	3.27 ± 0.07 ^ab^	117 ± 19.4
F40G	29.0 ± 6.51	197 ± 7.00	206 ± 11.6 ^bcd^	221 ± 48.3	3.23 ± 0.12 ^ab^	99.7 ± 24.7
F40S	33.3 ± 6.96	170 ± 7.69	220 ± 27.6 ^bc^	183 ± 34.7	3.20 ± 0.10 ^ab^	97.0 ± 11.0
F20	34.3 ± 1.45	220 ± 39.1	149 ± 16.4 ^de^	178 ± 12.9	2.87 ± 0.09 ^b^	97.3 ± 9.53
F20G	42.0 ± 6.35	184 ± 23.6	161 ± 27.5 ^cde^	186 ± 37.9	2.83 ± 0.20 ^b^	103 ± 9.84
F20S	32.7 ± 8.57	187 ± 55.9	138 ± 32.4 ^e^	246 ± 97.7	2.90 ± 0.15 ^b^	104 ± 25.6
Two-way ANOVA (*p* value)						
FM level	0.087	0.427	0.000	0.297	0.001	0.692
GABA	0.306	0.487	0.532	0.077	0.422	0.792
SB	0.781	0.362	0.358	0.408	0.486	0.808
FM × GABA	0.089	0.273	0.602	0.309	0.410	0.573
FM × SB	0.236	0.159	0.286	0.332	0.589	0.677

Values are the mean of triplicate groups and are presented as mean ± SD. Values with different superscripts in the same row are significantly different (*p* < 0.05). The lack of superscript letters indicates no significant differences among treatments. ^1^ Glutamic-oxaloacetic transaminase. ^2^ Alkaline phosphatase. ^3^ Total cholesterol. ^4^ Triglycerides. ^5^ Total protein. ^6^ Glucose.

**Table 5:** Biometric parameters of red seabream (*Pagrus major*) fed the nine experimental diets for 8 weeks.

	Condition Factor ^1^	Hepatosomatic Index ^2^	Viscerosomatic Index ^3^
F60	1.87 ± 0.02	1.20 ± 0.21	6.59 ± 0.69
F60G	1.77 ± 0.11	1.23 ± 0.27	6.73 ± 0.53
F60S	1.82 ± 0.09	1.22 ± 0.09	6.86 ± 0.10
F40	1.87 ± 0.03	1.25 ± 0.41	6.99 ± 0.18
F40G	1.77 ± 0.06	1.21 ± 0.32	6.86 ± 0.77
F40S	1.75 ± 0.06	1.22 ± 0.13	6.41 ± 0.41
F20	1.72 ± 0.05	0.95 ± 0.06	5.70 ± 0.36
F20G	1.78 ± 0.11	1.06 ± 0.14	6.30 ± 1.08
F20S	1.70 ± 0.06	1.22 ± 0.17	6.52 ± 0.50
Two-way ANOVA (*p* value)			
FM level	0.268	0.087	0.064
GABA	0.802	0.529	0.199
SB	0.402	0.413	0.039
FM × GABA	0.897	0.642	0.118
FM × SB	0.464	0.044	0.328

Values are the mean of triplicate groups and presented as mean ± SD. ^1^ Condition factor = weight (g) × 100/length^3^ (cm). ^2^ Hepatosomatic index = (liver weight (g)/fish weight (g) × 100. ^3^ Viscerosomatic index = (Viscera weight (g)/fish weight (g) × 100.

**Table 6:** Muscle proximate composition (%, wet basis) of red seabream (*Pagrus major*) fed the nine experimental diets for 8 weeks.

	Dry Matter	Protein	Lipid	Ash
F60	25.6 ± 0.68	20.0 ± 0.60	4.24 ± 0.36	1.29 ± 0.08
F60G	25.8 ± 0.44	19.9 ± 1.26	4.26 ± 0.22	1.29 ± 0.09
F60S	25.3 ± 0.34	19.4 ± 1.07	3.78 ± 0.90	1.40 ± 0.06
F40	25.0 ± 0.82	19.5 ± 0.91	2.65 ± 0.84	1.30 ± 0.06
F40G	25.2 ± 0.15	19.5 ± 0.35	3.03 ± 0.73	1.39 ± 0.01
F40S	24.9 ± 0.24	19.7 ± 0.39	3.64 ± 1.12	1.35 ± 0.05
F20	25.0 ± 0.61	20.1 ± 0.69	3.11 ± 0.67	1.42 ± 0.03
F20G	24.9 ± 0.27	20.4 ± 0.79	4.05 ± 0.67	1.35 ± 0.13
F20S	24.8 ± 0.32	19.6 ± 0.38	3.87 ± 0.46	1.34 ± 0.05
Two-way ANOVA (*p* value)				
FM level	0.095	0.706	0.213	0.708
GABA	0.630	0.879	0.389	0.865
SB	0.761	0.607	0.507	0.526
FM × GABA	0.644	0.971	0.754	0.563
FM × SB	0.960	0.835	0.610	0.235

Values are the mean of triplicate groups and presented as mean ± SD.

## Data Availability

The data that support the findings of this study are available from the corresponding author upon reasonable request.
